# Construction of artificial intelligence-assisted English learning resource query system

**DOI:** 10.3389/fpsyg.2022.970497

**Published:** 2022-10-12

**Authors:** Wenjing Yao, Ning Li

**Affiliations:** ^1^Department of Foreign Languages, Jinzhong University of Shanxi, Jinzhong, China; ^2^College of Foreign Studies, Liaoning University, Liaoyang, China

**Keywords:** artificial intelligence, English learning resources, query system, construction research, feedback mechanism

## Abstract

English has become an important tool for China's opening to the outside world and exchanges with other countries. More and more people have the motivation and requirements to learn English, but under the traditional English learning mode and traditional teaching mode, the cultivation of learners' autonomous learning habits is ignored. This article aims to study the construction of artificial intelligence-assisted English learning resource query system and establish the relevant feedback mechanism of retrieval. This article applies this mechanism to the retrieval of learning resources, so as to provide learners with the learning resources they really need and improve learners' learning efficiency. This article proposes to find the relevant knowledge points by extracting the knowledge points of the retrieval content. It realizes the query expansion based on knowledge and then realizes the expansion of retrieval results. It realizes the mapping of knowledge points on the retrieval content, the query and expansion of knowledge points, and the presentation of learning resources of the knowledge point index. It also uses the relevant feedback mechanism to adjust the retrieval results to meet the retrieval needs of learners. The experimental results show that the number of knowledge points can be increased to 2–4 times by query expansion based on English resources. Thus, the number of learning resources of search results can be increased to 3–10 times, the expansion of search results can be realized, and the overall recall will be greatly improved. In this article, the related methods of artificial intelligence are applied to the construction experiment of the English learning resource query system, which has a certain promotion effect on the construction of the system.

## Introduction

At present, English learning websites are full of money, and many websites have repeated contents. It is not easy to locate English learning websites with their own characteristics that meet the special needs of learners. Then it should realize that the key to attracting learners to stay for a long time and return again is to provide truly valuable information content with high accuracy, high credibility, and timely update. It focuses the main content of the web page on one aspect. It provides users with information references in this specific aspect. It makes it wonderful, rather than mixing all kinds of information together.

The network expands the function of computers in language teaching. As an open classroom, it enables foreign language learners to transcend the limitations of region, time, and space. It communicates with people from Britain and the United States in English. Learners can learn independently in this special classroom. Second, the network itself provides a treasure house of pure English resources. Its knowledge-based query expansion can explain and supplement the retrieved content through synonym expansion. By presenting the retrieval resources through the indexing of knowledge structure, it can mine the learning resources of the implicit index. It can reflect that there is an internal knowledge structure index between learning resources, and help learners better master the query content.

The innovations of this article are as follows: (1) this article uses the two parts of synonym expansion and knowledge index expansion of knowledge base to realize the relevant expansion of query, and integrates and confirms the two parts of expansion. (2) This article proposes a retrieval mechanism of relevant feedback on the retrieval results, which makes the retrieval results closer to the query requirements through relevant feedback.

## Related work

In addition to English, a large number of multilingual content on the Internet has given birth to the impulse to develop information retrieval systems that can cross-language boundaries. Bajpai et al., discussed the development process of a complete English to Hindi cross-language information retrieval system and the contribution of each component to the system. The experimental results obtained by him confirm that adding the component “analyzer” to the CLIR architecture improves the efficiency of disambiguation algorithm (Bajpai et al., 2018). The electronic dictionary system has become very important in today's society. Yong et al., conducted research on the intelligent English electronic dictionary system. He combined the advantages of the Internet of things to design and implement an electronic dictionary system (Yong, 2021). Although the software architecture in the process of dictionary application development is the research content, the development process of the electronic dictionary is not fully discussed. The key data of the training era involve the research and improvement of English learning ability. Kaleem et al., evaluated the importance and complexity of statements used in queries, which saves the work of the automated questionnaire system, including better skill testing and formal (Kaleem et al., 2017). Although the data direction is evaluated, there is no comprehensive test of candidates' skills. cross-language information retrieval (CLIR) system is convenient for users to query information in one language and retrieve relevant documents in another language. Thenmozhi et al., solved these problems by translating the Tamil language into English and retrieving English pages. He used the word sense disambiguation module to solve the ambiguity in Tamil queries (Thenmozhi and Chandrabose, 2018). However, the ambiguity between source query and translation query will reduce the performance of the system. Gao et al., extended and introduced KALM-QA (Gao et al., 2019) that answers more complex English problems. With the development of technology, it is easy to find information in news text. The purpose of fatmawati research is to analyze the implementation of common phrase indexing methods in information retrieval (Fatmawati et al., 2017). Although the study will be carried out in English news texts and implemented on prototypes, the degree of relevant of the generated documents is not determined. The growth of Internet-based and local applications does require that almost all Internet-based applications support global languages. Aadil and Asger has thoroughly investigated the machine transliteration models and machine learning methods (Aadil and Asger, 2017). Although machine transliteration and machine translation are used, support for local languages is not provided in all Internet-based applications.

From the relevant research on English learning resource retrieval, the construction of a knowledge ontology database, the establishment of a user interest model, situational awareness, and other means can meet the retrieval needs of specific situations to a certain extent. Building an ontology knowledge base is an effective means to realize semantic retrieval, which can realize the query expansion of retrieval content. This article is based on the construction of the knowledge base, and uses the knowledge structure in the knowledge base to realize the expansion based on knowledge, and establishes the index from learning resources to knowledge points. However, the methods related to learning resource retrieval basically analyze the needs of learners through modeling. It provides relevant learning resources. It lacks learners' judgment of retrieval results. The relevant feedback mechanism, which can be used as a means to optimize the retrieval results, has been well applied in many information retrieval systems, but it has not been well applied to learning resource retrieval.

This article uses the related methods of artificial intelligence, which is of great significance to the construction of the English learning resource query system. The innovation of this article is: using the synonym expansion of the knowledge base and the knowledge index expansion to realize the related expansion of the query, and to integrate and confirm the two parts of the expansion. And a retrieval mechanism is proposed to provide relevant feedback to the retrieval results, and the retrieval results can be closer to the query requirements through relevant feedback.

## Customer requirements and methods of English learning resource query system

### User on English learning resources website

The user base has been fully popularized, and the user's mentality has also changed. Due to the wide range of activities and many opportunities, people are unprecedentedly busy, time is precious, and there are more and more things to pay attention to. Too much information input is too late to digest, and the noisy life makes people more and more impatient (Berg and Aronoff, 2021). People are eager to organize complex information orderly and turn complex problems into simple ones. The idea of information construction can just guide how to display the content and characteristics of information in a clear structure to meet the needs of users. People exchange information and engage in various activities in virtual cyberspace, and the biggest consumption is time (Ramchandani et al., 2021).

Combined with the characteristics of English learning, this article divides users into non-professional and professional English learners. Most of the existing English Learning Websites target non-professional users. Because of its rich content and various forms, it can better meet the needs of users. At the basic level, as a supplement to literature-based learning materials, it provides users with richer resources to improve their basic English skills. But at the same time, literature resources, classrooms, and training courses also have great advantages, such as being more systematic, with special teachers to provide on-site guidance and supervision.

For deep users such as professional English learners, network resources have a more irreplaceable position. Their needs are often more specialized. In the learning process, they need to grasp the knowledge as a whole, from a single knowledge point to an understanding of relevant knowledge points (Islamova, 2019). For example, when learning an English article, students should not only master the vocabulary and grammar of the text itself, but also understand the relevant background knowledge, comments, and brief introduction of the author, and even extend to the study of other articles with the same subject and author. At this level, the network has incomparable advantages. Users can retrieve the required information with the click of the mouse. However, the huge information resources also make users face difficulties in choosing and obtaining. Therefore, it is necessary to build a good English learning website based on information.

### User information demand and information behavior

**Figure 1:**
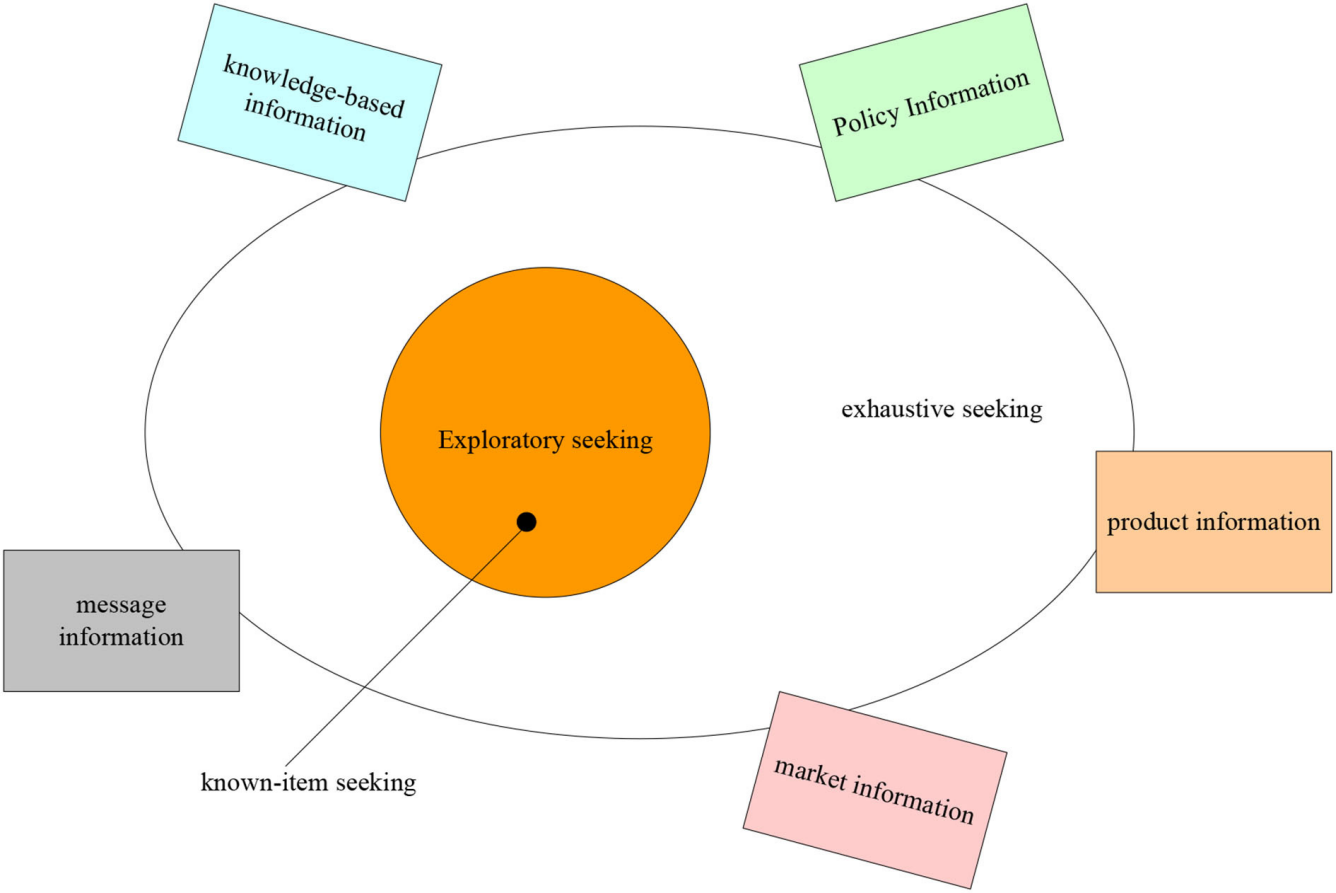
Three basic information needs of people.

Although English learning website users have different ages, occupations, interests, levels, and knowledge backgrounds, their information needs for learning materials are also different. Because there are great differences in the age, interest, knowledge structure, and professional background knowledge of English learning website users, they also show some differences in the content of information needs from the level and personality. It can be divided into two levels: high level and general level (Chen and Huang, 2021; Hong and Nam, 2021). In terms of content, high-level information needs are mostly innovative, verifiable, pioneering, and practical information. It can not only reflect the professional development characteristics of disciplines but also reflect the cross penetration, comprehensive and integrated development trend of disciplines. This kind of user requires strong academic and professional information. At the same time, it is also more standardized, such as users engaged in linguistics, translation, and literary research. They need more professional information and higher academic authority. People engaged in it need computer English, those engaged in law need legal English, and those engaged in trade need business English. Users with general level information needs are those who want to practice the basic skills of English listening, speaking, reading, writing, and translation. What they need is some practical, novel, and effective learning materials (Yong, 2021). The three basic information needs of people are shown in Figure 1.

As shown in Figure 1, corresponding to the above three information needs, users' information query behavior also has three basic forms: searching, such as inputting retrieval formula in the search engine; browsing, such as viewing information from one link to another by clicking the mouse; asking is asking others for help on the Internet, such as asking others questions through email, chat system, etc. Users' information needs are closely related to information behavior. Different information needs partially determine different information query behaviors.

### Neural network algorithm

Due to the uneven amount of questions students do, some students do more and some students do less. There are also differences in the focus of teaching resources and teaching requirements between colleges and universities. Moreover, when English teaching resources diagnose students, they often think intuitively. Under the characteristics of English resources, in order to make the system diagnosis more accurate and personalized, this module adopts the combination of the neural network and expert system. It makes intuitive thinking and logical thinking complementary. It transforms the experience and knowledge of English experts into a nonlinear problem. It enables the diagnosis process to realize the nonlinear mapping of input / output. The final diagnosis result is obtained by the BP neural network.

#### Activation function

The judgment of students' mastery of knowledge points is actually the fault diagnosis of students' knowledge points. In the field of fault diagnosis, the forward multilayer network is the most used and effective. In neural networks, even if a linear function can be selected theoretically, such as the identity function f (x) = x, the nonlinear sigmoid function is usually selected (Dombrovan et al., 2021).


(1) $$\begin{array}{l} f\left(x\right)=simg\left(x\right)=\frac{1}{1+e^{\left(-x\right)}} \end{array}$$

**Figure 2:**
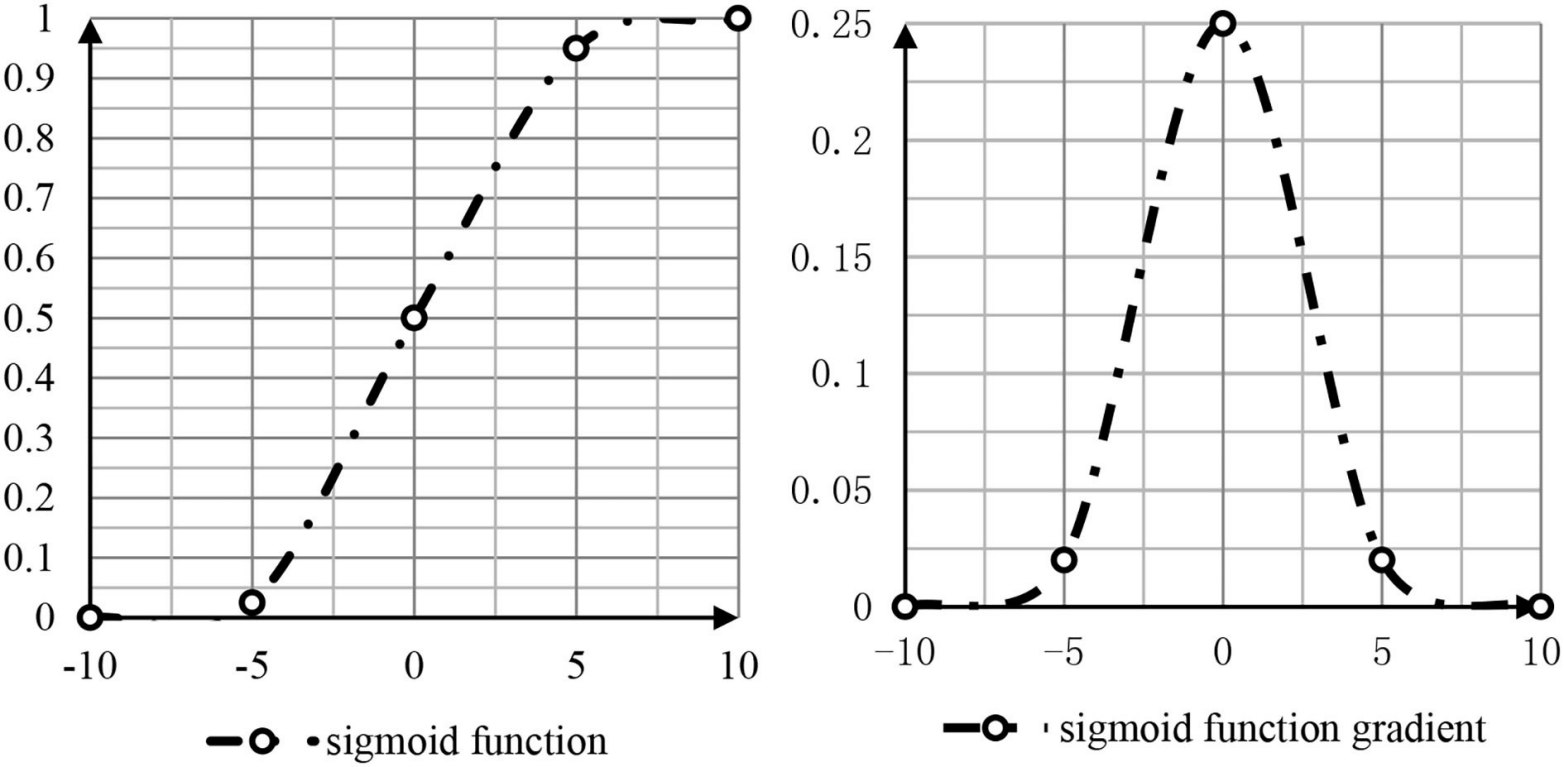
Sigmoid function and gradient.

The nonlinear sigmoid function and function gradient are shown in Figure 2.

As shown in Figure 2, the function value of sigmoid is between 0 and 1. The closer the function value of the independent variable is to 0, the faster the function value changes. The larger the absolute value of the function changes, the slower the absolute value of the function changes (Nie, 2021). In addition to the nonlinear sigmoid function, the second commonly used is the hyperbolic tangent function tahn:


(2) $$\begin{array}{l} \text{tahn}\left(x\right)=\frac{e^{x}-e^{-x}}{e^{x}+e^{-x}} \end{array}$$

In addition, the hard limiting function plus the skewness function can also be used to activate the function:


(3) $$\text{hardlimit}(x)=\{\begin{array}{ll} 1,x\geq 0 & \;\\ 0,x\leq 0 & \; \end{array}$$

(4) $$\text{ramp}(x)=\{\begin{array}{ll} 1,x\geq 1 & \;\\ x,-1\leq x\leq 1 & \;\\ -1,x\leq -1 & \; \end{array}$$

At the same time, there are two good choices, namely, the positive linear element ReLu and the leakage correction linear element.


(5) ReLU(x)=max(0,x)

(6) LReLU(x)={1,x≥0 ax,x≤0 

**Figure 3:**
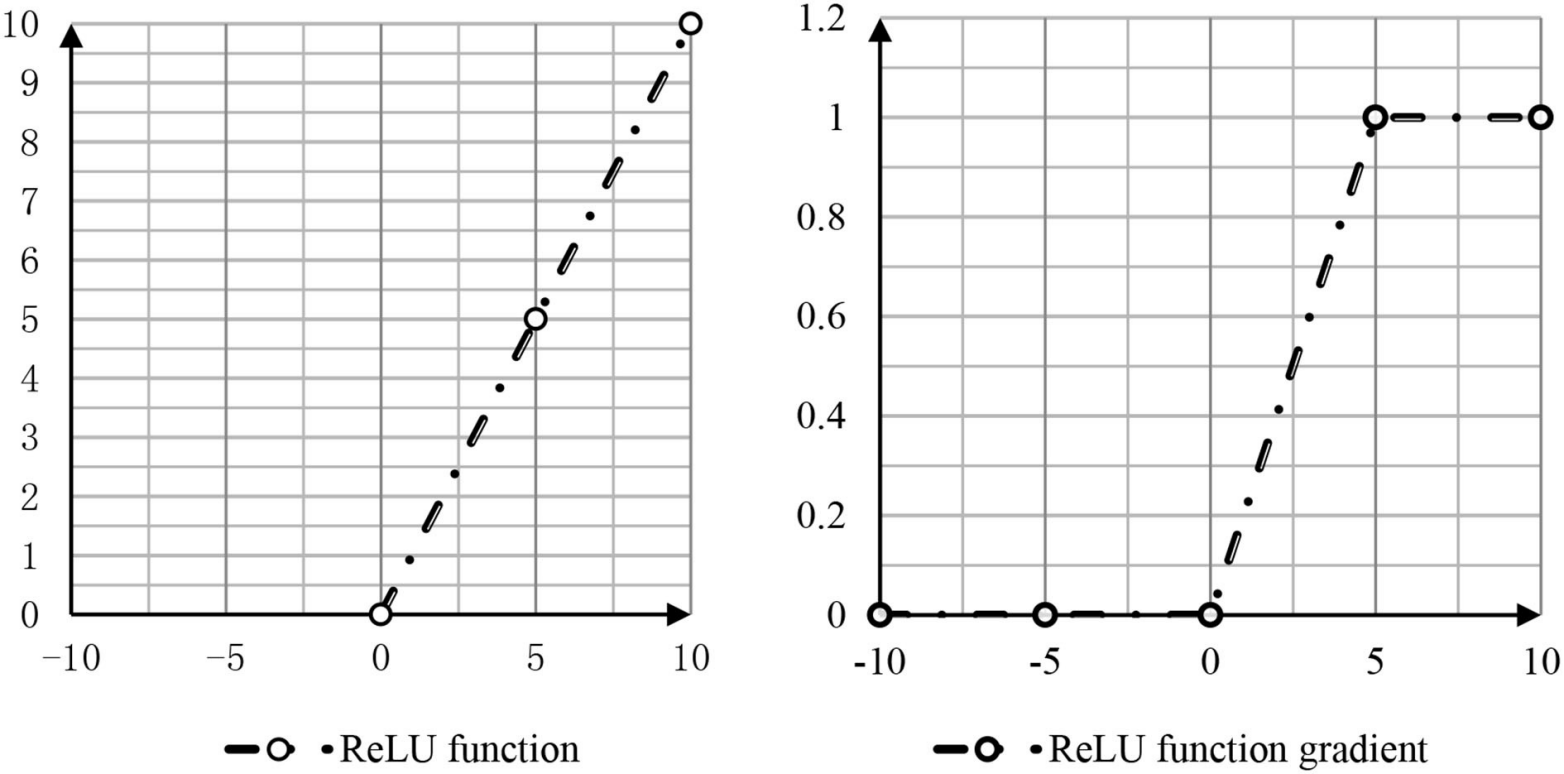
ReLu function and gradient.

The function and gradient of ReLu are shown in Figure 3.

As shown in Figure 3, in the classical structure, the activation function using the neural network is called rectified function. In order to alleviate the “dead zone” phenomenon, the researchers adjusted the part of x < 0 in the ReLu function to ψ(*x*) = δ*x, where a is a smaller positive number of the order of 0.01 or 0.001 (Narayanan and Mathew, 2021). This new activation function is called leaky ReLu:


(7) $$\text{Leaky ReLU(x)}=\{\begin{array}{ll} x(x)\geq \text{0)} & \;\\ \delta \;\cdot \;x(x\leq 0) & \; \end{array}$$

For the setting of δ in randomized ReLU, its value is uniformly distributed in the training phase, and it is designated as the distribution expectation (1+u) /2 of the uniform distribution in the testing phase.


(8) $$\text{Randomized ReLU(x)}=\{\begin{array}{ll} \text{x(x}\geq \text{0)} & \;\\ \delta \;\cdot \;x(x\leq 0) & \; \end{array}$$

Among them


(9) $$\begin{array}{l} \delta^{{}^{\prime }}\approx U\;\left(l,u\right),l\leq u,and\;l,u\in \left[0,1\right] \end{array}$$

However, the exponential operation slightly increases the amount of calculation:


(10) $$\text{ELU(x)}=\{\begin{array}{ll} x\text{ x}\geq \text{0} & \;\\ \beta^{.}(exp(x)-1)\text{x}<0 & \; \end{array}$$

#### Cyclic neural network

**Figure 4:**
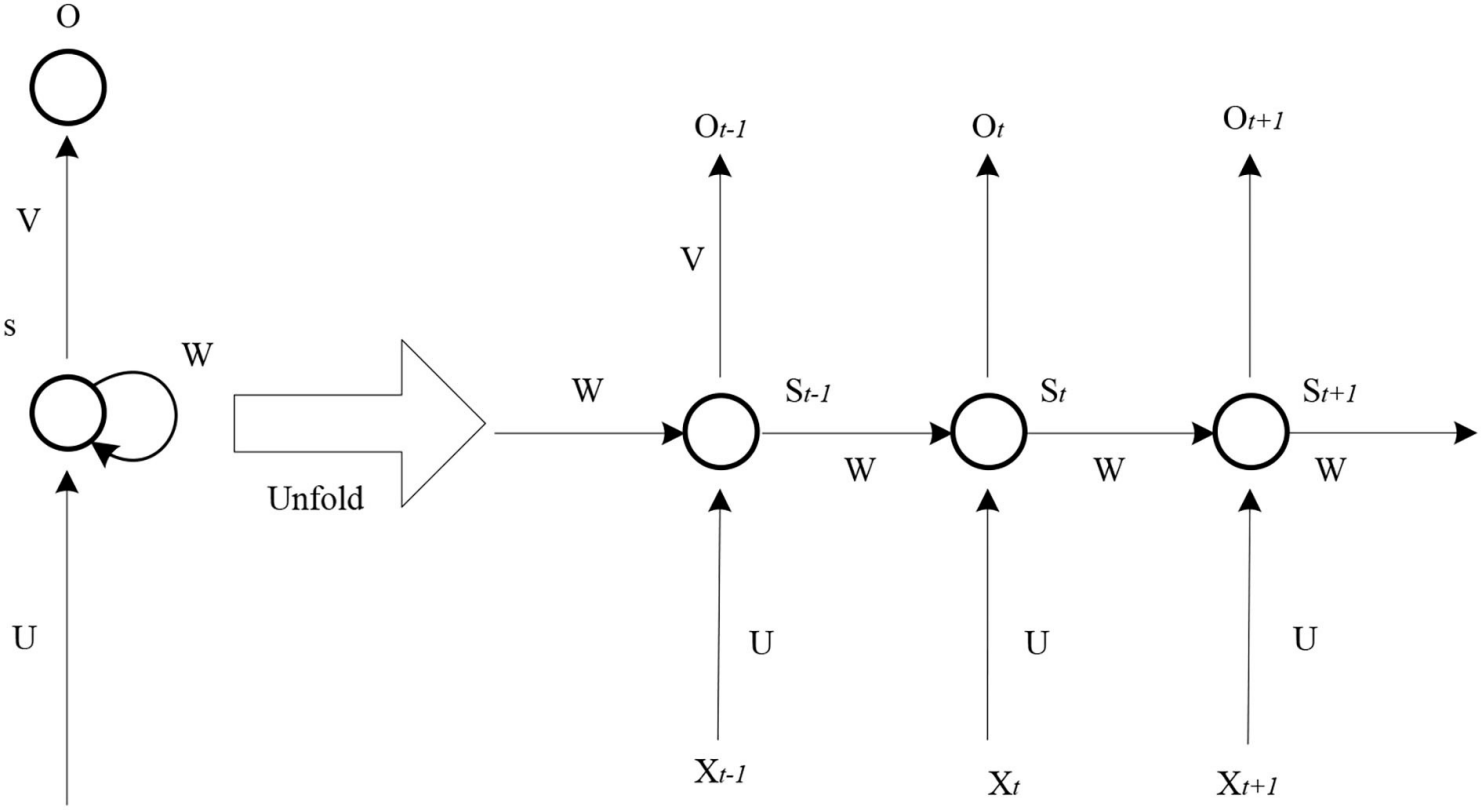
Schematic diagram of the simple structure of the cyclic neural network model.

When the research field is switched to natural language processing, it is necessary to use the cyclic neural network model for modeling (Bolu et al., 2020). In the application of language, there is a great connection between the preceding and the following. When a word in a sentence is removed, people usually choose the appropriate word to fill in the air in combination with the experience of the previous and subsequent text (Fitria, 2020). The structure of the cyclic neural network model is simple, and the schematic diagram is shown in Figure 4.

As shown in Figure 4, it is difficult to solve such problems in the traditional linguistic model. How to make the computer understand “language” is a difficult thing. Therefore, it proposes a recurrent neural network (RNN). Its main feature is that the network model has memory. In theory, RNN can trace any number of words forward.

The information of RNN at a certain time can contain the information of the previous time. It can find the sequence information of the previous and subsequent text and the semantic information of the text. Ordinary neural networks can only deal with short sequences of fixed length, and there is no relationship between the information of the previous window and the information of the next window in the process of moving the window.

At time t, the network has an input x, and the neuron state of the network *S*_*t*_ at this time can be expressed by formula (11):


(11) $$\begin{array}{l} S_{t}=f\left({ux}_{t}+{ws}_{t-1}\right) \end{array}$$

That is, sofmax processing is conducive to error backpropagation. The output *O*_*t*_ expression of the network at time t is (12):


(12) $$\begin{array}{l} O_{t}=soft\max\left({vs}_{t}\right) \end{array}$$

RNN model is mainly used to solve time series problems, so the loss of all times must be included in the calculation of model loss (Yuan et al., 2020). The loss function of the model at time t is shown in formula (13):


(13) $$\begin{array}{l} {loss}_{t}=-\left[y_{t}\ln\;\left(o_{t}\right)+\left(y_{t}-1\right)\ln\;\left(1-o_{t}\right)\right] \end{array}$$

*y*_*t*_ is the real label value input at time t, and *o*_*t*_ is the predicted value of the model. RNN is prone to gradient disappearance and explosion. In the process of model training, when the gradient is large, the function will directly skip the optimal solution, making the training unable to converge (Meng, 2020).

### Based on the attention pooling BiGRU model

**Figure 5:**
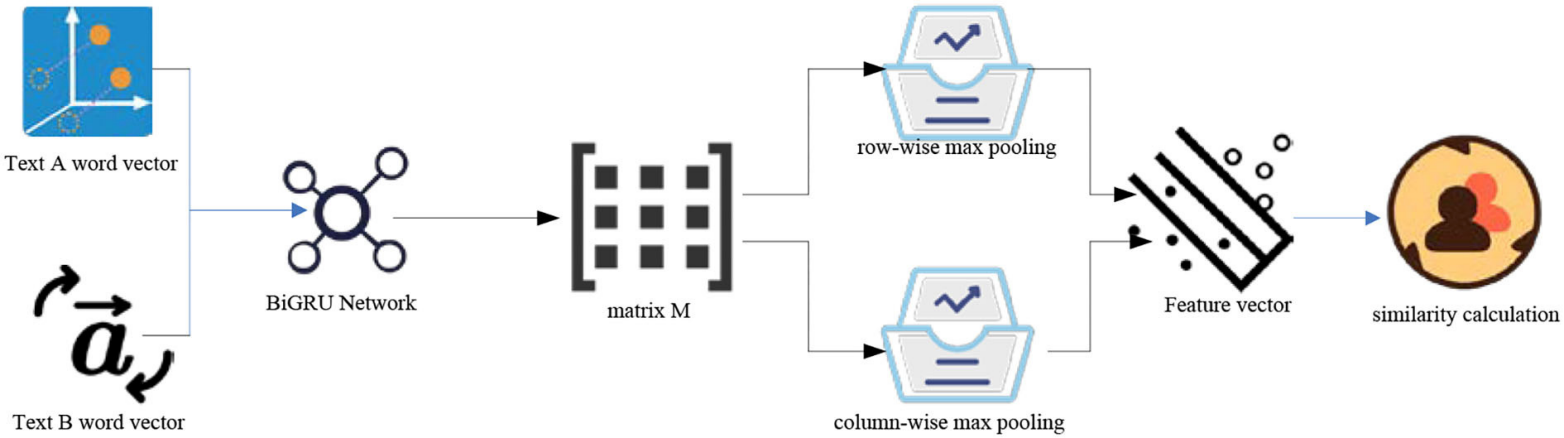
Structure of attention pooling model.

Attention pooling is introduced. The main feature of attention pooling is to construct an attention pooling matrix M for text matrix A and text matrix B processed by the neural network (Do et al., 2021; Flint et al., 2022). Matrix M generates the maximum pool vector of a row and the maximum pool vector of a column, respectively. Maximum pooling preserves the most significant features of the vector and reduces the computational complexity of the model (Liang, 2022; Pantanowitz, 2022). The model structure is shown in Figure 5.

As shown in Figure 5, the two vectors are normalized and multiplied by a and B to obtain the features finally integrated into the attention pool (Saura et al., 2022a). The matrix M can be obtained by formula (14).


(14) $$\begin{array}{l} M=\tanh\left(A^{T}UB\right) \end{array}$$

u is the parameter matrix obtained by neural network learning, which can be adjusted and optimized in the process of model training. M actually multiplies the text pair matrix indirectly through the parameter matrix. The converted attention vector is obtained (Liu, 2021). Taking the attention vector δ^*a*^ as an example, the ith element of δ^*a*^ is expressed as formula (15):


(15) $$\begin{array}{l} {\left[\delta^{a}\right]}_{i}=\frac{e^{\left[m^{a}\right]i}}{\sum_{1<l<t}e^{\left[m^{a}\right]l}} \end{array}$$

t is the text length. Multiply the original matrix by the normalized attention weight vector to obtain the final text pair representation vector:


(16) $$\begin{array}{l} r^{a}=A\delta^{a} \end{array}$$

(17) $$\begin{array}{l} r^{b}=B\delta^{b} \end{array}$$

Finally, the similarity between text pairs is calculated by cosine, which can be expressed as:


(18) $$\begin{array}{l} \cos\left(\theta \right)=\frac{<r^{a},r^{b}>}{\left||r^{a}||||r^{b}|\right|} \end{array}$$

In the model based on attention pooling, its input is the word vector matrix obtained by two text pairs (Saura et al., 2022b). Because the context of the text is related, we can't ignore the backward semantics and only focus on the meaning of a single word.

### Distance measurement method

When it is necessary to measure the similarity between samples, the usual method is to calculate the distance between text vectors (Abdi and Valentin, 2007; Saura et al., 2021a). There are many methods to calculate the distance. Choosing different methods will affect the accuracy of the judgment of the result.

#### Euclidean distance

Euclidean distance uses the most intuitive method to calculate the distance, that is, to calculate the real distance between two points in space, as shown in formula (19):


(19) $$\begin{array}{l} D=\sqrt{\sum_{i=1}^{n}{\left(x_{i}-y_{i}\right)}^{2}} \end{array}$$

#### Manhattan distance

It represents the sum of the distances between two points in the direction of the coordinate axis (Saura et al., 2021b). The calculation is as follows (20):


(20) $$\begin{array}{l} D=\sum_{i=1}^{n}|x_{i}-y_{i}| \end{array}$$

#### Hamming distance

It represents the number of different data at the same position of the sequence. The calculation formula is as follows (21):


(21) $$\begin{array}{l} D=\sum_{i=1}^{n}x_{i}⊕y_{i} \end{array}$$

## Construction of artificial intelligence-assisted English learning resource query system

### Construction principles of English query system

#### User-oriented principle

To build an efficient English learning website, we must first consider the problems from the perspective of users. Website designers must always realize that although the construction is aimed at the construction of information content such as English learning resources, it is carried out for users. Therefore, we should organize, manage and provide information according to users' views and behavior habits. The behavior habits of English learning resource users in the network environment are different from those in the traditional environment. For example, there are obvious differences between reading learning materials on the website and reading paper documents. When reading paper materials, users' reading habits are linear reading word by word and sentence by sentence, reading one text at a time without interruption, paying equal attention to each word, and inputting other information such as music, radio, television, and conversation at the same time. In the network environment, different users have many different reading methods for the same material text. Reading is not necessarily continuous reading from left to right or from front to back. Users usually skip reading. As long as people click the mouse, different readers will skim this part and not that part. And reading is accompanied by sound, image, and other information. In a relaxed reading environment, readers unknowingly enter new websites or texts from different parts. In addition, due to the different expression forms of information on the website, unlike the traditional black and white text, the expression forms of each word from beginning to end are not very different. So they get different attention from users.

#### Content first principle

**Figure 6:**
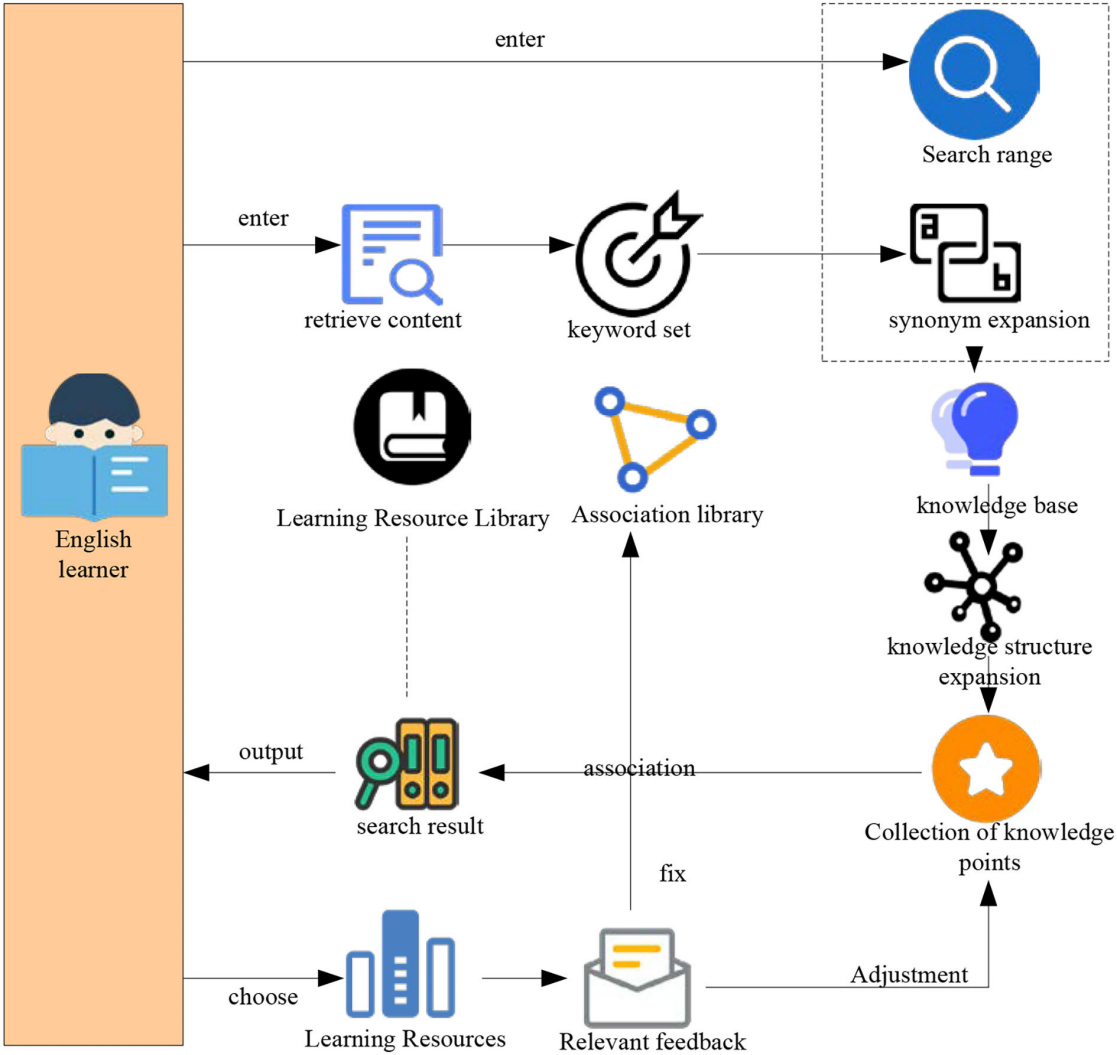
Overall research route.

After the Internet entered the field of English education, great changes have taken place in the way English teachers and learners teach and learn. Once users encounter any questions and need any information, they will first think of an online query. Many times, users visit the site with a certain purpose and specific content. The information needed by English learning resources website users is usually comprehensive, centralized, stable, fast and accurate, knowledge unit, hierarchical and personalized. With a large number of English resources and the need for resource content, there is a need for information construction. The object of information construction of English learning resources is English learning resources. Therefore, resource content is the leading and core of information construction, and everything serves the content. In addition, many English learning resources, learners' valuable time, and reading on the computer screen is easy to cause visual fatigue and other reasons, so users usually browse the content on the web instead of reading carefully. Therefore, this article proposes a knowledge-based query expansion and relevant feedback retrieval method. The overall research route of knowledge-based query expansion and relevant feedback retrieval is shown in Figure 6.

As shown in Figure 6, in the field of education, the index between knowledge points cannot be reflected at the simple text level. It makes it difficult for the retrieval system to expand the retrieval of knowledge points and keywords from the semantic level. Second, at present, it uses learners' relevant feedback information to adjust the query, which makes the query closer to learners' query intention. This is also one of the problems that the retrieval system is difficult to solve.

### Implementation of English learning resource retrieval

In the retrieval of learning resources, the traditional keyword-based retrieval method has two defects: First, the retrieval results often only meet the requirements of learners literally. Actual needs often deviate from learners. The second is to take the learning resource database as the retrieval object, which needs to match each learning resource, resulting in low retrieval efficiency. The disadvantages of limited depth and low efficiency of learning resource information retrieval systems hinder the utilization and sharing of learning resources.

To realize knowledge-based learning resource retrieval, we first need to prepare a corpus. It uses natural language processing technology and related algorithms to build the index of learning resources to knowledge points. It indexes knowledge points and learning resources. Then it determines the knowledge system through the query input, calculates the similarity between the retrieval content and the knowledge points, and extracts the knowledge points. It takes the extracted knowledge points as query-related knowledge points. It further presents the learning resources indexed by knowledge points to learners in the reverse order.

#### Index construction based on LSA

The purpose of constructing a learning resource index is to extract useful information from unstructured learning resources. According to the concept and semantics of knowledge points in the knowledge base, the mapping relationship between learning resources and knowledge points is established. Thus, it can use the structured knowledge in the knowledge base to expand the semantics of the retrieval keywords and carry out the subsequent retrieval work based on this.

The mapping relationship between learning resources and knowledge points needs to use natural language processing-related technologies. It mainly includes word segmentation, removing stop words, and so on. Word segmentation refers to dividing a paragraph or sentence into several words or words according to syntactic logic and common word combination habits: removing stop words refers to removing commonly used cohesive words, meaningless words, or specific words that need to be filtered according to the stop word list after word segmentation. In the selection of natural language processing technology, this section uses Jieba word segmentation to extract keywords. Jieba word segmentation provides a variety of methods to segment text to varying degrees, and it can also obtain the weight of keywords to construct the vector model. Some fields describing information in the knowledge base and learning retrieval base need to be filtered, so it is necessary to establish a stop vocabulary based on the knowledge base and learning resource base. This can effectively improve retrieval efficiency.

**Figure 7:**
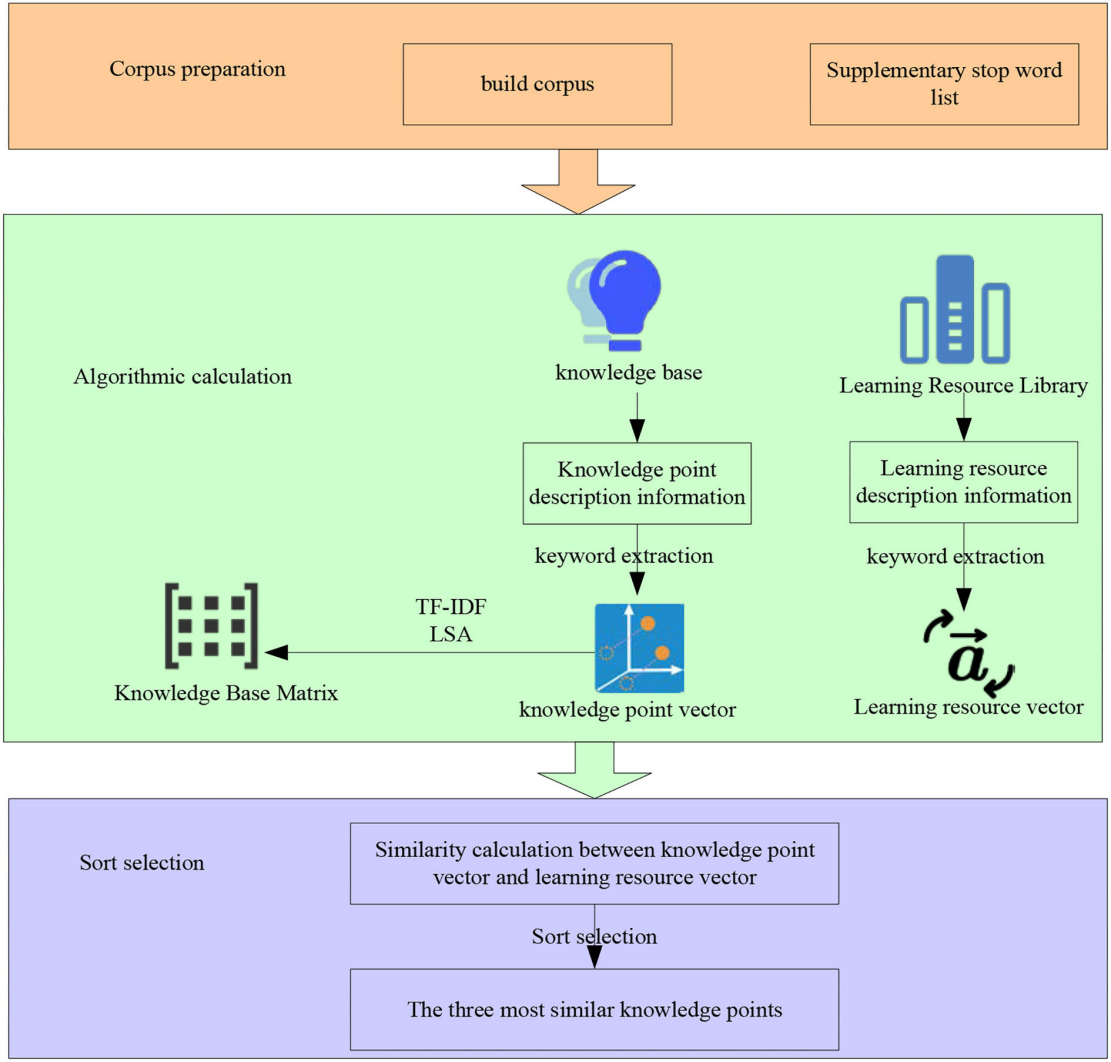
Construction process of knowledge-based learning resource index.

The vector space model is used in the model. The vector space model considers the weight and can describe the correlation between knowledge points and learning resources. The construction process of the knowledge-based learning resource index is shown in Figure 7.

As shown in Figure 7, first, it takes the description information of all knowledge points in the knowledge base as the training corpus. The TF-IDF method is used to calculate the keyword weight of each knowledge point description information to form a knowledge point vector. It also uses LSA to decompose the knowledge base matrix by SVD. For each resource in the learning resource database, word segmentation and remove the stop words in the learning resource description information, extract keywords and transform them into learning resource vectors. It uses the cosine similarity calculation method to calculate the similarity between each resource and all knowledge point vectors. It selects the three knowledge points with the greatest similarity as the knowledge to be indexed. In an implementation, this section uses the Gensim Library in Python language to realize the indexing of learning resources to knowledge points. The good scientific computing power of Python language has been widely used in artificial intelligence and other related fields. Gensim is a natural language processing library that can be called directly by python. It supports a variety of model algorithms including TF-IDF and LSA.

#### Query knowledge point extraction

Generally, the retrieval system directly matches the retrieval content with the resource database, mainly because the resources in the resource database are not systematic. It establishes a knowledge base to express the same learning resources and establishes an index from learning resources to knowledge points. Then we can find the learning resources that learners want according to the knowledge points in the knowledge base. Therefore, in the retrieval process, only the knowledge points of the retrieval content need to be extracted. After obtaining the relevant knowledge points, we can obtain the learning resources related to the retrieval content.

In order to realize the knowledge extraction of retrieval content, this article makes the knowledge point extraction results of retrieval content meet the needs of learners through two parts. First, this article determines the knowledge system that learners need to retrieve. All knowledge points in the knowledge system are the set of knowledge points to be selected for knowledge extraction. Then, through the method of natural language processing, it uses the vector space model to calculate the similarity between the retrieval content and each knowledge point in the set of knowledge points to be selected. It arranges the similarity between the knowledge points and the retrieval content in the descending order and takes the knowledge points of topn as the knowledge extraction result.

The accuracy of the retrieved relevant knowledge points depends entirely on the query. The actual situation is that different learners describe the same word differently. Often, documents related to query requests cannot be retrieved due to different words. In addition to retrieving relevant knowledge points, learners are often interested in the relevant knowledge points in the knowledge structure. It needs to take other knowledge points related to this knowledge point in the knowledge structure as relevant knowledge points. In order to solve these problems, this section proposes a knowledge-based query expansion method. The knowledge-based query expansion is divided into two parts: one is to expand the query by using synonyms to supplement and explain the query. One part extends the query based on the knowledge structure and provides knowledge-related extension based on the knowledge structure. After implementing the two-part extension, it needs to fuse the two-part extension with different weights, and finally provide the query extension results to learners. Learners confirm the knowledge-based query expansion and present relevant learning resources according to the learners' confirmed relevant knowledge points.

### Relevance feedback mechanism based on the knowledge

**Figure 8:**
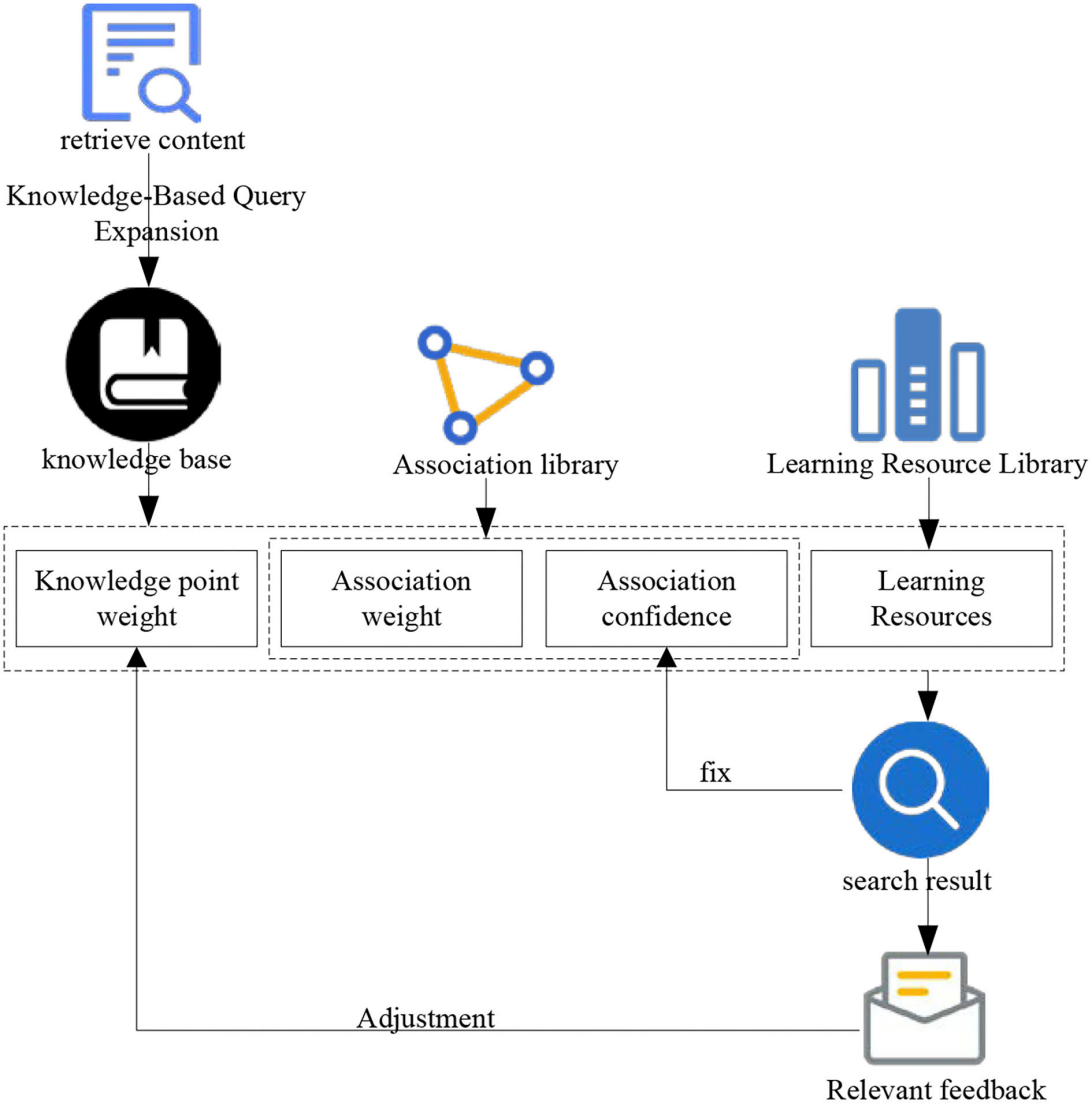
Knowledge-based feedback mechanism.

The essence of the knowledge-based relevant feedback mechanism is to use the user's relevant feedback to optimize the retrieval. The difference is that knowledge-based relevant feedback is not directly used to modify the query, but to adjust the query-related knowledge points. Its working principle is that learners submit queries representing their query needs to the retrieval system. The retrieval system makes an initial query and returns the query-related knowledge points. It returns the collection of learning resources through the index of knowledge points and learning resources. It is usually arranged in the descending order according to the correlation between learning resources and queries, and then learners judge the correlation between learning resources and queries and point out which are relevant or irrelevant. The process of relevant feedback can continue until learners get more satisfactory learning resources. The relevant feedback mechanism based on knowledge is shown in Figure 8.

As shown in Figure 8, the learner judges whether each learning resource retrieved is relevant or irrelevant. Each relevant judgment of learners will generate a new record in the retrieval feedback database. The record is composed of the unique identification of knowledge points, the unique identification of learning resources, the unique identification of users and whether it is relevant or not. Statistics from different dimensions can produce different feedback effects. The use of these statistical information can help better retrieval of knowledge-based learning resources. In this section, knowledge-based relevant feedback retrieval is mainly reflected in two parts: one part is to count the relevant feedback information from different learners to the learning resource index of the same knowledge point. It proposes the concept of index confidence, which modifies the index based on natural language processing technology. The other part counts the relevant and irrelevant learning resources that the learner thinks through the feedback of a single learner. It adjusts the weight of knowledge points related to queries under relevant learning resources and irrelevant learning resources, so as to adjust the retrieval results.

### Database design

The MySQL database is used in database selection. It designs a learner information table for storing learner-related information, a knowledge point table for storing knowledge point information, a learning resource table for storing learning resource information, an index table for storing index information of knowledge points and learning resources, and a retrieval feedback table for storing retrieval feedback information.

In the design of table, it strictly follows the database design specification to ensure the consistency and integrity of data. The specific data sheet is designed as follows:

(1) The learner information table is used to store the basic information of learners, mainly including: learner name, level, and login password. The specific structure is shown in Table 1.

**Table 1:** Learner information.

**Serial number**	**Name**	**Describe**	**Type**
1	user_ id	learner id	int[11]
2	username	learner name	varchar[10]
3	password	current password	varchar[20]
4	field	field of study	enum
5	grade	grade of study	enum
6	subject	Subject	enum

(2) The knowledge point table is used to store the basic information and knowledge structure of the knowledge point, mainly including: the name of the knowledge point, the description information of the knowledge point, the grade discipline of the knowledge point, and the parent knowledge node of the knowledge point. The specific structure is shown in Table 2.

**Table 2:** Knowledge points.

**Serial number**	**Name**	**Describe**	**Type**
1	knowid	knowledge point id	int[11]
2	knowledge_ title	Knowledge point name	varchar[50]
3	pre_ knowid	parent node id	int[11]
4	description	Description	varchar[200]
5	keywords	keywords	varchar[50]
6	field	field of study	enum
7	grade	grade of study	enum
8	subject	Subject	enum

(3) Learning resource table is used to store basic information such as learning resource name, learning resource type, description information, grade, and discipline. The specific structure is shown in Table 3.

**Table 3:** Learning resources.

**Serial number**	**Name**	**Describe**	**Type**
1	r_id	learning resource id	int[11]
2	URL	Url address	varchar[20]
3	res_type	Resource Type	enum
4	Resource Name	Resource Name	varchar[50]
5	describe	Description	varchar[200]
6	res_ _difficulty	degree of difficulty	enum
7	site	field of study	enum
8	grade	grade of study	enum
9	Subject	Subject	enum

(4) The retrieval feedback table is used to store the feedback information of learners on the retrieval results. The specific structure is shown in Table 4.

**Table 4:** Search feedback.

**Serial number**	**Name**	**Describe**	**Type**
1	id	record id	int[11]
2	knowid	knowledge point id	int[11]
3	r_ _id	resource id	int [11]
4	user_id	learner id	decimal
5	content	retrieve content	varchar[50]
6	relation	relevant or irrelevant	enum
7	update_time	update time	datat ime

## Experimental on the construction of English learning resource query system

### Knowledge-based query expansion experiment

**Figure 9:**
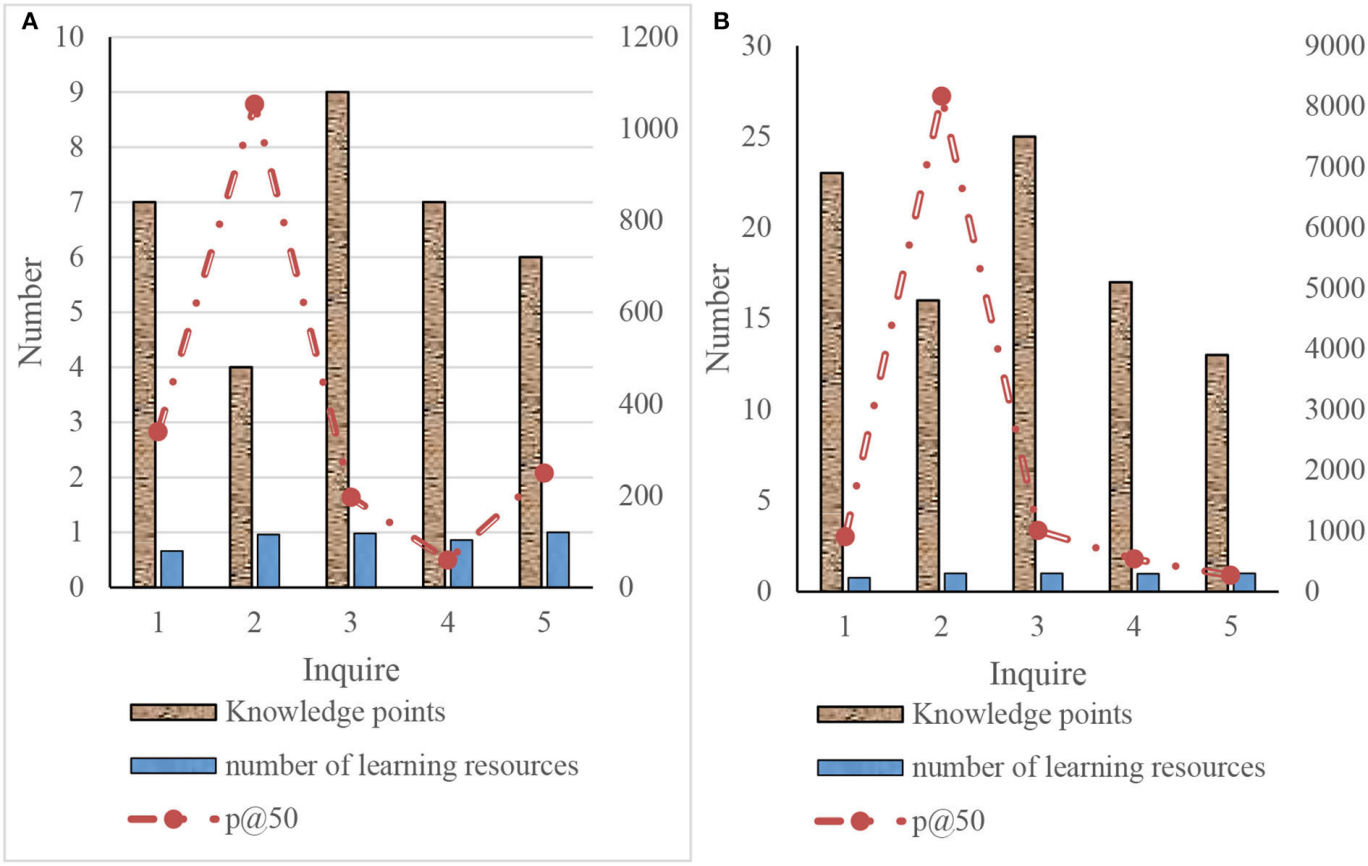
Experimental results of query expansion retrieval. **(A)** Knowledge-based retrieval and **(B)** knowledge-based query extended retrieval.

Because the retrieval implementation of this article is based on a large-scale corpus, the recall is difficult to measure. The recall can only be expressed indirectly by the number of returned knowledge points and learning resources. In the measurement of precision, it is used in large-scale corpus P@5O. It calculates the accuracy of the first 50 learning resources returned. This article uses the five queries established to count the number of returned knowledge points and learning resources and calculate the accuracy of the first 50 learning resources. The specific results are shown in Figure 9.

As shown in Figure 9, through the comparison of the retrieval results of knowledge-based retrieval and knowledge-based query expansion, it can be concluded that:

(1) From the perspective of a full search, through knowledge-based query expansion, it can increase the number of knowledge points to 2–4 times the original. Thus, the number of learning resources of search results can be increased to 3–10 times, the expansion of search results can be realized, and the overall recall will be greatly improved. In the fifth query, when the number of knowledge points is doubled, the number of returned resources does not change greatly. The possible reason is that the number of resources of the extended knowledge point index is very small. The knowledge point to the learning resource index constructed by semantic analysis has a small number of learning resources.

(2) From the perspective of accuracy, P@50 has been improved through knowledge-based expansion. The lower the P@50 of knowledge-based learning resources is, the higher the improvement of the P@50 of the retrieved learning resources after knowledge expansion. However, the final P@50 still largely depends on knowledge-based learning resource retrieval. For the fifth query, the P@50 of both methods is 1. It shows that when constructing the index of learning resources to knowledge points through semantic analysis, the accuracy is high, but the number is not enough. This also confirms why the number of learning resources has not changed greatly after the expansion of knowledge points.

### Relevance feedback experiment based on English resources

#### Experimental purpose

To verify the feasibility of the knowledge-based relevant feedback method, it should also be divided into two parts: using the relevant feedback mechanism to adjust the query and maintain the index. Index maintenance requires a large number of learners to participate in feedback in order to get more accurate results. Due to the limitations of conditions, the experiment cannot be carried out. Therefore, when the default index confidence is 0.5, the experiment using the relevant feedback mechanism to adjust the query is carried out.

#### Experimental data

In the experiment of knowledge-based query expansion, the relevant feedback is given to the retrieval results. It also judges the correlation between the first 50 learning resources in the search results and the query and inputs the same query again on this basis. It carries out multiple rounds of feedback and compares the changes in P@50 under different rounds of feedback. In order to avoid the mutual influence of retrieval feedback from two different methods, experiments need to be carried out with different users and independent environments.

#### Experimental results

**Figure 10:**
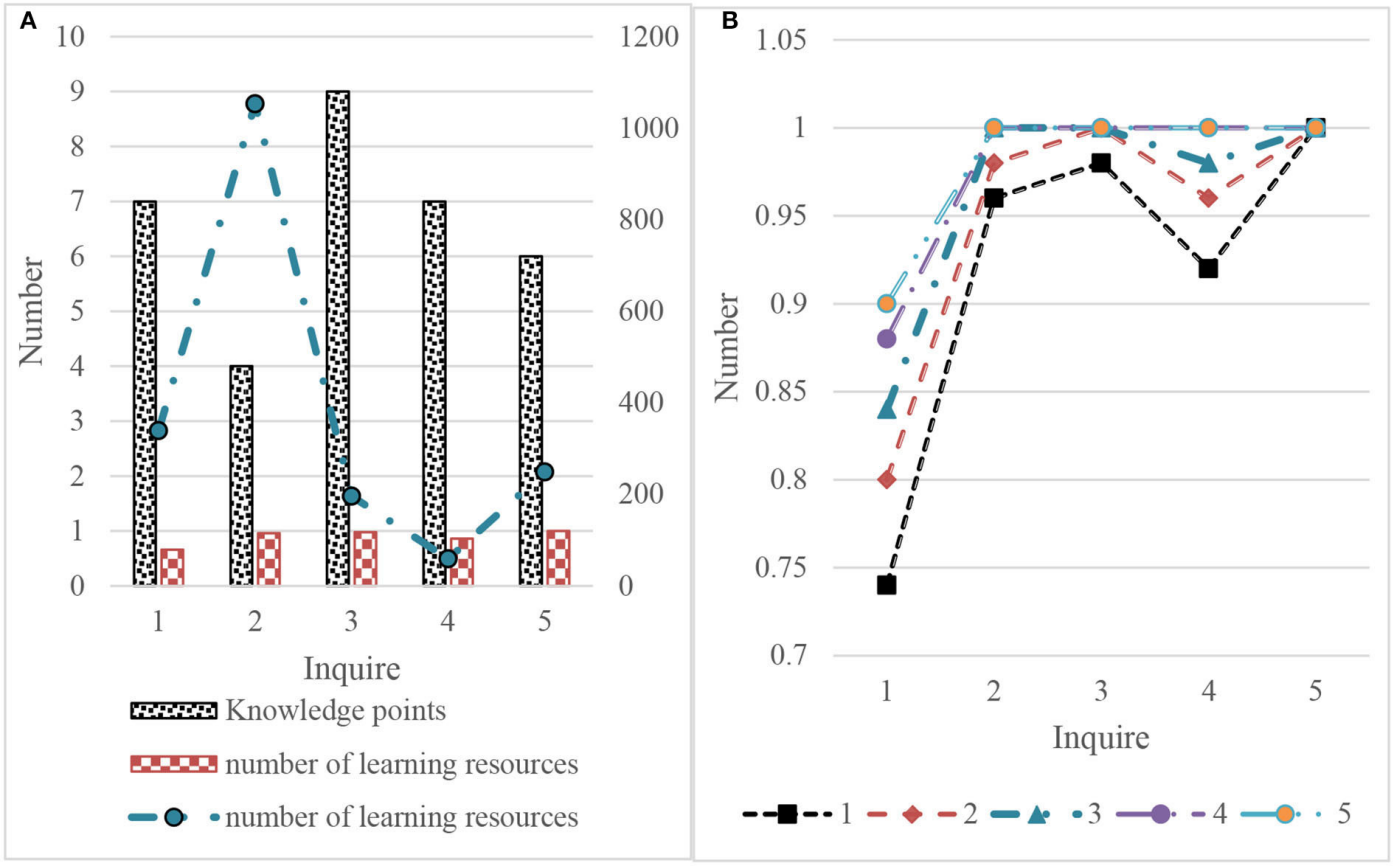
Experimental results of relevant feedback retrieval based on knowledge. **(A)** Knowledge based retrieval. **(B)** Feedback rounds.

In the experiment of knowledge-based query expansion, this article adopts the same query. It carries out the relevant feedback on the retrieval results of knowledge-based retrieval and knowledge-based query expansion retrieval, respectively. In order to measure the effectiveness of relevant feedback, it carries out five rounds of feedback to see the changes in P@50 in each round. The experimental results are shown in Figure 10.

**Figure 11:**
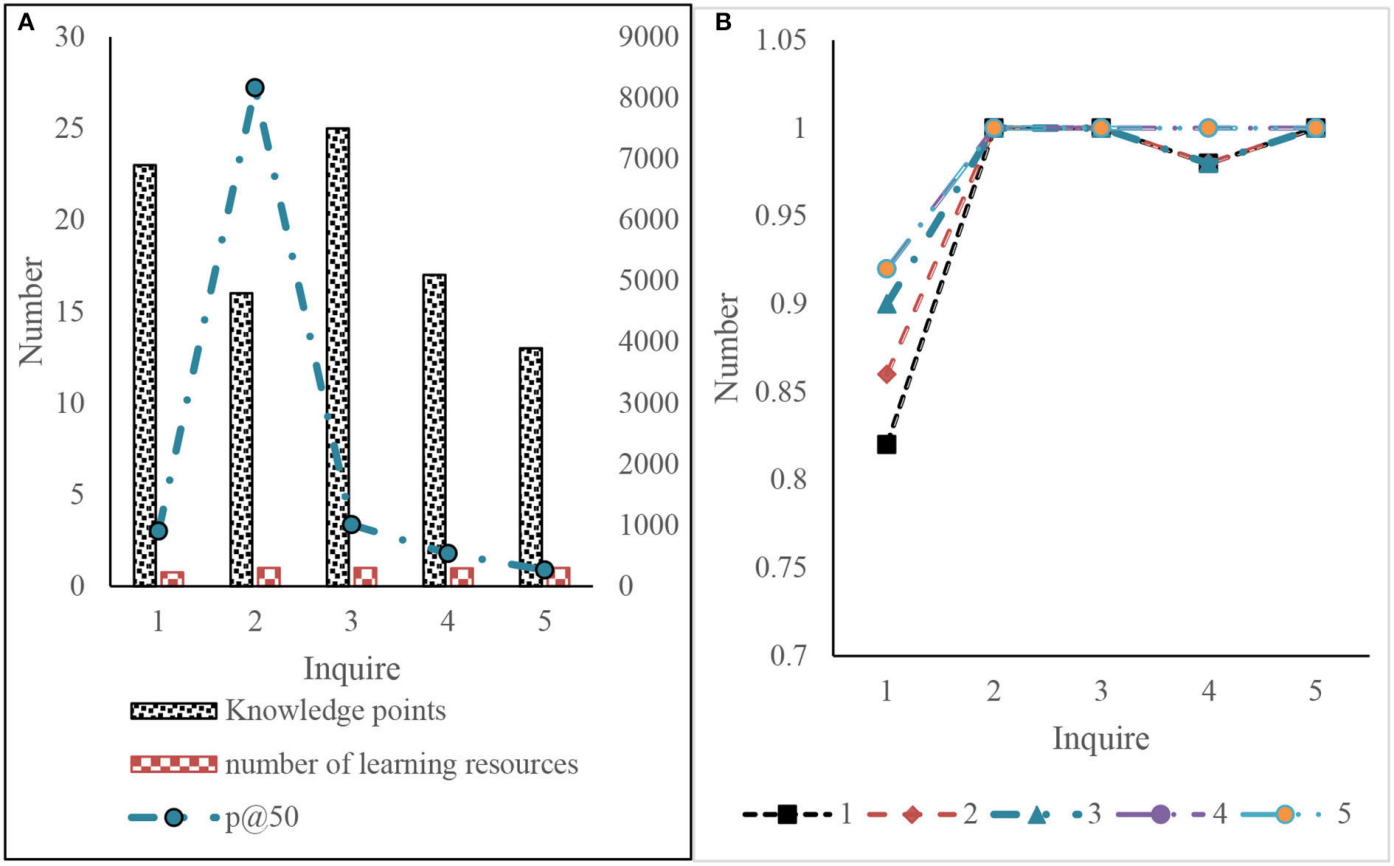
Experimental results of knowledge-based query expansion and relevant feedback. **(A)** Knowledge based query extended retrieval **(B)** feedback rounds.

The following results can be obtained according to the initial feedback in Figure 11, which will not affect P@50 as shown in Figure 10. Therefore, the relevant feedback can still be used when the initial accuracy is very high. For the case where the initial P@50 is high but not l, the use of the relevant feedback can improve P@50, but multiple rounds of feedback may be required. For the case of low initial P@50, the first few rounds of relevant feedback can be greatly improved, and the later promotion becomes slow or even impossible. This may be related to the number of relevant learning resources. If the number of relevant learning resources is insufficient, there will be learning resources related to insufficient queries in the first 50 query results.

## Discussion

This article mainly uses the related methods of artificial intelligence to conduct experimental analysis on the construction of the auxiliary English learning resource query system. This article first analyzes and introduces the related algorithms of artificial intelligence. Then, take the construction of the query system as the research object, and find out that the query accuracy of the query system is very high, and the relevant feedback can still be used. The full text is based on the artificial intelligence algorithm to study the construction of the auxiliary English learning resource query system. This is not only a further expansion of the research field of research methods, but also an in-depth discussion of the research on the learning resource query system.

## Conclusion

The lack of information construction of English learning resources website will lead to the reduction of website effectiveness and ease of use, and affect users' satisfaction with the website. There are not only quantitative factors such as the number of clicks, the number of views, and the number of search results but also qualitative factors such as the simplicity of information organization, the clarity of navigation, the ease of learning of the search system, and so on. From the depth of retrieval, the method of establishing a knowledge base to express learning resources is conducive to the sharing and reuse of learning resources. It can understand the knowledge semantics of the retrieval content input by learners through the retrieval system, and the retrieval of learning resources can be transformed into the retrieval of knowledge points. According to the knowledge points, it can get the learning resources related to the retrieval needs. From the perspective of retrieval efficiency, on the premise of the huge learning resource base, online retrieval system needs to give retrieval results in real-time. If all resource databases are used as retrieval objects, the efficiency will be very low, which cannot meet the needs of learners, resulting in the loss of interest in learning. Knowledge points can be used to describe learning resources, and the number of knowledge points is far lower than that of learning resources. Knowledge points are used to represent learning resources and the resource index of knowledge points can be established offline. In this way, the efficiency of retrieval is avoided because of a large number of calculations. Because the experimental data of this experiment come from actual analysis, the objectivity is strong, the data source is more realistic, and the reliability of the experimental results is high. However, due to the existence of some defects in the experiment, the experiment needs to be further improved.

## Data availability statement

The original contributions presented in the study are included in the article/supplementary material, further inquiries can be directed to the corresponding author.

## Author contributions

WY put forward research ideas and editing. NL design research scheme, collecting, and analyzing data. Both authors contributed to the article and approved the submitted version.

## Funding

This work was supported by the Teaching Reform and Innovation Project of colleges and universities in the Shanxi Province Research on the Construction and Application of Dynamic Discourse Corpus from the perspective of Multimodality (MDA) (No. J2021635) from the Shanxi Provincial Education Department. It was supported by the Teaching Reform and Innovation Project of Jinzhong University Research on the teaching resource database construction and application of project-based foreign language flipped classroom based on VR (No. Jg201922) from the Jinzhong University. It was supported by the program of High-quality Sharing course Cross-cultural communication (No. KC2020003) at Jinzhong University in 2020. This work was supported by the University-Industry Collaborative Education Program Construction of Practice Base for Innovative and Compound Talents of Business English under the Background of New Liberal Arts (Project Number: 202102107018).

## Conflict of interest

The authors declare that the research was conducted in the absence of any commercial or financial relationships that could be construed as a potential conflict of interest.

## Publisher's note

All claims expressed in this article are solely those of the authors and do not necessarily represent those of their affiliated organizations, or those of the publisher, the editors and the reviewers. Any product that may be evaluated in this article, or claim that may be made by its manufacturer, is not guaranteed or endorsed by the publisher.
